# Neural Response to Biological Motion in Healthy Adults Varies as a Function of Autistic-Like Traits

**DOI:** 10.3389/fnins.2017.00404

**Published:** 2017-07-14

**Authors:** Meghan H. Puglia, James P. Morris

**Affiliations:** Department of Psychology, University of Virginia Charlottesville, VA, United States

**Keywords:** social perception, fMRI, individual differences, autism quotient, biological motion

## Abstract

Perception of biological motion is an important social cognitive ability that has been mapped to specialized brain regions. Perceptual deficits and neural differences during biological motion perception have previously been associated with autism, a disorder classified by social and communication difficulties and repetitive and restricted interests and behaviors. However, the traits associated with autism are not limited to diagnostic categories, but are normally distributed within the general population and show the same patterns of heritability across the continuum. In the current study, we investigate whether self-reported autistic-like traits in healthy adults are associated with variable neural response during passive viewing of biological motion displays. Results show that more autistic-like traits, particularly those associated with the communication domain, are associated with increased neural response in key regions involved in social cognitive processes, including prefrontal and left temporal cortices. This distinct pattern of activation might reflect differential neurodevelopmental processes for individuals with varying autistic-like traits, and highlights the importance of considering the full trait continuum in future work.

## Introduction

Successful navigation of a complex and dynamic social world requires the ability to perceive, interpret, and respond appropriately to social cues. As such, human perceptual systems are specialized to preferentially detect and attend to social information including the movements of animate beings, known as biological motion (Johnson, 2006). Research using point-light displays, which capture movement of the major joints, indicates that the dynamics of biological motion alone are sufficient for the automatic perception of an animate being (Johansson, 1973). Rather than requiring other perceptual cues, humans are able to recognize not just animacy, but even emotions (Atkinson et al., 2004) and intentions of others (Manera et al., 2010) based solely on the dynamics of these impoverished stimuli.

Biological motion perception can be differentiated from perception of other forms of coherent motion and mapped to specialized neural systems. For example, posterior superior temporal sulcus (pSTS) is an important region for perception of biological motion and plays a critical role in understanding motion within a social context (Allison et al., 2000). Sensitivity to biological motion emerges early in development (Bardi et al., 2011) and may serve as a precursor to subsequent social-cognitive abilities (Pavlova, 2012).

Autism spectrum disorder (ASD), a neurodevelopmental disorder defined by deficits in social and communication abilities and restricted, repetitive behaviors and interests, is often associated with differences in biological motion perception. When compared to control groups, individuals with ASD fail to orient to (Klin et al., 2009; Annaz et al., 2012) or show decreased visual sensitivity for biological motion stimuli (Blake et al., 2003; Kaiser et al., 2010a). During biological motion perception, autistic individuals often show hypoactivation of pSTS (Herrington et al., 2007; Freitag et al., 2008; Kaiser et al., 2010b; Ahmed and Vander Wyk, 2013) and regions of prefrontal cortex (PFC; Kaiser et al., 2010b; Koldewyn et al., 2011). However, group-level differences are not always consistent (e.g., Murphy et al., 2009; Saygin et al., 2010), which might reflect the vast heterogeneity in social cognitive abilities within both clinical and non-clinical populations. For example, it has been shown that even within autistic groups, visual sensitivity to biological motion varies as a function of severity of the disorder (Blake et al., 2003). Therefore, considering the autism spectrum as a continuous measure may better enable the detection of meaningful biomarkers associated with this important social perceptual ability.

It is increasingly understood that the traits associated with ASD are not limited to diagnostic categories, but are normally distributed within the general population (Baron-Cohen et al., 2001). Unaffected individuals with a high number of autistic-like traits also fail to automatically attend to (van Boxtel and Lu, 2013) or show decreased visual sensitivity for human biological motion stimuli (Kaiser and Shiffrar, 2012; Miller and Saygin, 2013). Converging evidence suggests that autistic traits are specifically associated with difficulties with global, holistic interpretations of human motion, rather than perceiving coherent motion cues *per-se* (van Boxtel et al., 2016). For example, higher autistic-like traits are associated with difficulties detecting human but not object or animal motion (Kaiser and Shiffrar, 2012), the direction that a point-light walker is facing, but not walking (Miller and Saygin, 2013), and whether dynamic stimuli depict humans interacting (van Boxtel et al., 2016).

Non-clinical autistic-like traits are also associated with differences in brain structure and function in regions important for perceiving and understanding human motion (von dem Hagen et al., 2011; Nummenmaa et al., 2012; Anderson et al., 2013; Thurman et al., 2016). For example, Anderson et al. (2013) found a negative association between a measure of social impairment associated with autism and activation to biological motion in frontal and partial regions in a non-clinical sample. However, these authors only considered traits in the social domain, and did not report descriptive statistics for this measure making it difficult to assess what range of the spectrum was captured in this study.

The current study adds to this growing body of literature suggesting neural differences in global biological motion processing across the autistic-trait continuum by adopting several important methodological considerations: (1) we include a large sample that displays high variability in autistic-like traits to identify how neural response to biological motion varies across the non-clinical autism spectrum; (2) we employ a whole-brain exploratory approach to avoid an overly modular view of the brain and examine whether autistic-like traits are associated with recruitment of differential neural networks beyond those canonically associated with biological motion perception; (3) we use a measure of autistic traits that considers both social and non-social behaviors to explore whether specific aspects of the autistic phenotype are differentially associated with neural response; and (4) we utilize a passive viewing paradigm. While previous studies used forced-choice discrimination paradigms (e.g., Thurman et al., 2016), it is well-known that instructing participants to identify an animate entity within a dynamic display biases viewing behavior and neural response toward this end (Stanley et al., 2010; Zwickel et al., 2012; Lee et al., 2014; Gowen et al., 2016). Therefore, we expect a passive viewing paradigm to evoke differences in participants' spontaneous tendency to globally perceive or interpret stimuli as biological, thus optimizing our ability to detect individual differences in neural response.

## Methods

### Participants

Fifty-three participants without history of medical or neurological disorders participated in the present study as part of a larger imaging study aimed at examining individual differences in social perceptual processes. All participants provided written informed consent for a protocol approved by the University of Virginia Institutional Review Board and were paid $50. Two participants were excluded from analysis: one participant did not wear corrective lenses during scanning and reported difficulty seeing the stimuli, and one participant was excluded due to an fMRI data collection error. Fifty-one healthy adults (26 males) aged 18 to 25 (*M* = 20.69, *SD* = 1.54) years were included in the final analysis.

### Biological motion perception task

Participants passively viewed twelve 24-s blocks of point-light displays alternating between biological (six blocks) and scrambled (six blocks) motion while undergoing fMRI. Point-light stimuli depicted an adult male performing salient social-interactive actions such as waving and playing peek-a-boo (Klin et al., 2009; Kaiser et al., 2010b). Critically, to avoid priming biases related to expectations of biological motion (Stanley et al., 2010; Zwickel et al., 2012; Lee et al., 2014; Gowen et al., 2016) participants were told that they would watch a series of short videos, but were given no additional details about the content of the videos and not explicitly informed that stimuli might depict biological motion.

### Quantification of autistic-like traits

Participants completed the Autism Spectrum Quotient Questionnaire (AQ), a 50-item self-report measure of traits and behaviors associated with autism (Baron-Cohen et al., 2001). Participants are asked to rate the degree to which they agree or disagree with statements regarding behaviors and preferences. Approximately half of the items are reverse scored (r). Total scores can range from 0 to 50 and each of the five subscores can range from 0 to 10. Higher scores reflect endorsement of more autistic-like characteristics. Items assess social skill [e.g., (r) “I find social situations easy”], communication (e.g., “When I talk, it isn't always easy for others to get a word in edgeways”), attention switching [e.g., (r) “I find it easy to do more than one thing at once”], attention to detail (e.g., “I tend to notice details that others do not”), and imagination [e.g., (r) “If I try to imagine something, I find it very easy to create a picture in my mind”]. The AQ is among the most widely used measures to quantify autistic-like traits (Ruzich et al., 2015; Landry and Chouinard, 2016) and offers several advantages over other continuous measures of autistic-like traits: The AQ (1) was specifically designed to quantify autistic-like traits in the general population, rather than familial occurrence among unaffected relatives or as a diagnostic screening measure; (2) includes questions about preferences to circumvent difficulties with self-awareness of one's behaviors or abilities; (3) assesses both social and non-social behaviors; and (4) is freely available and does not require specific administrative qualifications, making it broadly accessible to researchers. AQ scores for the present sample are detailed in Table 1.

**Table 1:** Descriptive statistics for AQ scores.

**AQ Score**	**Min**	**Max**	**Mean**	***SD***	***W***	***P***
Total	4	32	15.73	5.37	0.98	0.434
Social skill	0	7	1.76	1.83	0.84	<0.0001
Attention switching	0	9	4.12	1.99	0.96	0.073
Attention to detail	0	10	5.65	2.22	0.97	0.241
Communication skill	0	7	2.22	1.87	0.89	<0.0001
Imagination	0	5	1.98	1.39	0.92	0.002

*Min, minimum; Max, maximum; SD, standard deviation; W, Shapiro–Wilk test of normality; P, significance of Shapiro-Wilk test of normality*.

### Image acquisition and preprocessing

Scanning was performed at the University of Virginia on a Siemens 3 Tesla MAGNETOM Trio high-speed imaging device equipped with a 12-channel head-coil. High-resolution T1-weighted anatomical images were first acquired using Siemens' magnetization-prepared rapid-acquired gradient echo (MPRAGE) pulse sequence with the following specifications: echo time (TE) = 2.53 ms; repetition time (TR) = 1,900 ms; flip angle (FA) = 9°; field-of-view (FOV) = 250 mm; image matrix = 256 mm × 256 mm; slice thickness = 1 mm; 176 slices. Whole-brain functional images were then acquired using a T2^*^ weighted echo planar imaging (EPI) sequence sensitive to blood oxygenation level dependent (BOLD) contrast with the following specifications: TE = 40 ms; TR = 2,000 ms; FA = 90°; FOV = 192 mm; image matrix = 64 mm × 64 mm; slice thickness = 3.5 mm; slice gap = 22%; 148 volumes of 28 slices co-planar with the anterior and posterior commissures. Stimuli were presented with Psychophysics Toolbox for MATLAB (Brainard, 1997) using an LCD AVOTEC projector onto a screen located behind the subject's head and viewed through an integrated head-coil mirror.

Data preprocessing was carried out using FEAT (FMRI Expert Analysis Tool) Version 6.00, part of FSL (FMRIB Software Library; Smith et al., 2004). Motion was assessed by center of mass measurements (BXH/XCEDE Tools, version 1.8.16, Bioinformatics Information Research Network) to ensure that no participants had >1 mm deviation in the x-, y-, or z-dimensions. The following pre-statistics processing was applied: motion correction using MCFLIRT (Jenkinson et al., 2002); slice timing correction; non-brain removal using BET (Smith, 2002); spatial smoothing using a Gaussian kernel of 4.0 mm full width at half maximum to reduce noise; grand-mean intensity normalization of the entire 4D dataset by a single multiplicative factor; high-pass temporal filtering (Gaussian-weighted least-squares straight line fitting, with sigma = 50.0 s). Additionally, each functional volume was registered to the participant's high resolution anatomical image, and then to FSL's standard Montreal Neurologic Institute (MNI 152, T1 2 mm) template brain using FSL's linear registration tool (FLIRT; Jenkinson et al., 2002). Registration from high resolution structural to standard space was then further refined using FSL's non-linear registration, FNIRT (Andersson et al., 2007a,b).

### fMRI analysis

Data analysis was conducted using FEAT (FMRI Expert Analysis Tool) Version 6.00, part of FSL (Smith et al., 2004). At the subject level, time-series statistical analysis was carried out using FSL's improved linear model (FILM) with local autocorrelation correction (Woolrich et al., 2001). Regressors for each condition (biological and scrambled motion) were modeled by convolving the time course with a double-gamma hemodynamic response function (HRF), adding a temporal derivative and applying temporal filtering. A biological>scrambled motion contrast was conducted, and the contrast of parameter estimates (COPE) from this analysis for each individual was carried forward to higher-level analysis.

At the group level, all analyses were conducted with a standardized approach using FSL's local analysis of mixed effects (FLAME) stage 1 (Beckmann et al., 2003; Woolrich et al., 2004; Woolrich, 2008). A recent study using a mass empirical analytic approach concluded that FSL's FLAME stage 1 cluster-wise correction outperforms that of other popular fMRI software packages and provides a valid method for familywise error correction with error occurring at or below the expected rate (Eklund et al., 2016). All *Z* (Gaussianised T/F) statistic images were thresholded using clusters determined by Z > 2.3 (*p* < 0.01) and a corrected cluster significance threshold of *p* < 0.05 (Worsley, 2001). The minimum significant cluster size under this p-threshold is 197 voxels.

We first conducted a one-sample *T*-test to determine the main effect of the task, which identifies regions that are similarly activated to the task contrasts across individuals. Mean lower-level activation for each participant was entered into the model, and contrasts testing group biological>scrambled motion and biological<scrambled motion activation were computed. This analysis was used to ensure that, when considered as a whole group, the present sample activated regions expected to be involved in biological motion perception.

We next conducted an exploratory whole-brain analysis to determine the relationship between task-specific BOLD response and the occurrence of autistic-like traits. Two regressors were included in the model, group mean and mean-centered total AQ score, and contrasts testing for positive and negative linear relationships between AQ score and biological>scrambled BOLD activation were computed. Because one participant's total AQ score was >3 *SD* above the mean, we took additional steps to ensure that outliers did not bias this model. Specifically, we applied FSL's automatic outlier de-weighting algorithm to this analysis, which identifies and de-weights outliers within the fMRI data (Woolrich, 2008). Clusters that survived correction were registered to subject space and mean *Z*-statistic values were extracted for each participant from these clusters. We then tested for outliers by ensuring that the absolute value of both mean and median standardized residuals of the AQ model for each cluster was <3 for each data point. Finally, we tested for data points with undue influence on the overall model for each cluster using Cook's distance (*D*) > 1 (Cook and Weisberg, 1982) as criteria. No outliers or influential points (all *D* < 0.39) were detected.

To illustrate significant effects, clusters that survived correction were registered to subject space, and mean *Z* statistic values were extracted for each participant from these clusters and plotted against their AQ score.

Both neural systems involved in biological motion perception (Anderson et al., 2013) and the occurrence of autistic-like traits (Baron-Cohen et al., 2001) have previously been shown to differ across sexes. We therefore also conducted an AQ × sex interaction whole-brain analysis to determine whether the linear relationship between total AQ score and BOLD response differs across sexes. For each sex, group mean, and mean-centered total AQ score were included as regressors in the model, and contrasts testing for differences in linear relationship between AQ and BOLD response were computed.

Finally, to determine whether any particular subscore of the AQ was driving the effects seen in the exploratory analysis conducted with total AQ score, we conducted separate whole-brain analyses assessing the relationship between task-specific BOLD response and each AQ subscore (social skill, communication, attention switching, attention to detail, and imagination). The AQ is a broad measure assessing multiple domains associated with the autism spectrum. This exploratory analysis provides more specific information about behavioral differences that might drive individual differences in the neural systems involved in social perceptual processes. Group mean and mean-centered AQ subscore were included as regressors in each model, and contrasts testing for positive and negative linear relationships between AQ subscore and biological>scrambled BOLD activation were computed. Analyses with significant clusters surviving correction were compared in spatial location and cluster extent to the clusters identified by the analysis completed with Total AQ score.

## Results

### Main effect of biological motion perception task

**Figure 1:**
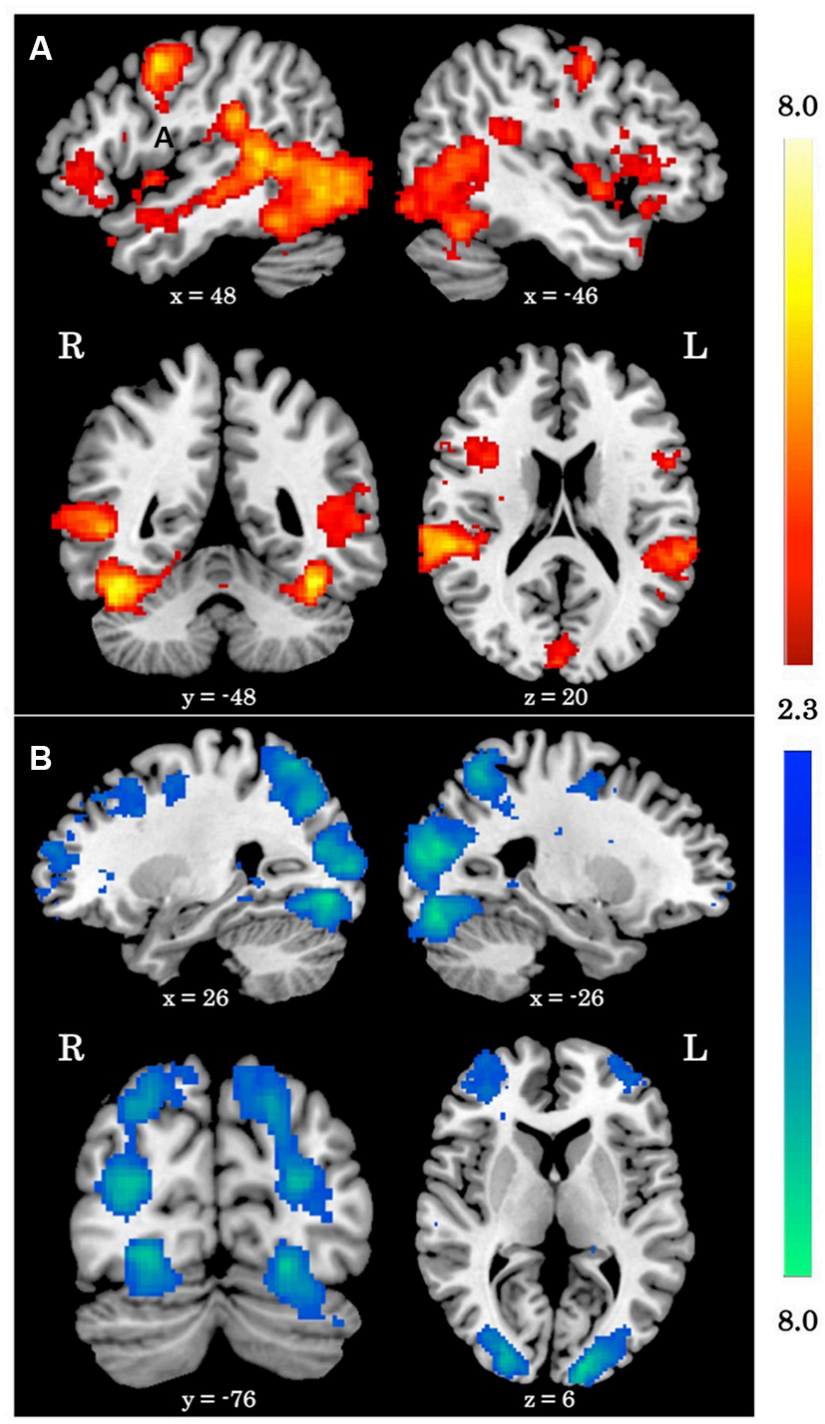
Main effect of biological motion perception task. **(A)** *Z* statistic maps showing significant clusters of activation for the biological>scrambled motion contrast. **(B)** *Z* statistic maps showing significant clusters of activation for the biological<scrambled motion contrast. All coordinates are in MNI space.

When averaging across the whole group, regions typically involved in biological motion perception, including bilateral pSTS and bilateral fusiform gyrus were preferentially activated by the biological>scrambled motion contrast in a manner consistent with past research (see Figure 1). Local maxima statistics for the task main effect analysis are detailed in Table 2.

**Table 2:** Local maxima statistics for clusters showing a main-effect of task.

**Contrast**	**Anatomical region**	**Hem**	***x***	***y***	***z***	***Z***	***k***
Biological>Scrambled motion	Temporal cortex	L	−40	−48	−20	6.55	4,208
	Temporal cortex	R	42	−48	−22	6.47	7,404
	Primary motor cortex	R	52	2	46	6.02	615
	Lateral occipital cortex	L	−28	−100	−8	5.47	208
	Supplementary motor cortex	B	−6	−8	62	4.74	420
	Lingual gyrus/Cuneus	B	0	−90	22	4.55	886
	Insula/Inferior frontal gyrus	L	−24	−2	−20	4.55	1,564
	Orbitofrontal cortex	R	54	34	2	4.18	472
	Putamen	R	22	6	2	3.83	210
	Inferior frontal gyrus	R	36	12	22	3.76	275
Biological<Scrambled motion	Occipital cortex	B	−18	−88	−16	8.62	13,927
	Dorsolateral prefrontal cortex	R	48	20	38	4.83	1,857
	Rostrolateral prefrontal cortex	R	28	58	18	4.12	1,428
	Posterior cingulate cortex	B	4	−28	38	3.67	261
	Middle frontal gyrus	L	−28	2	54	3.47	207
	Rostrolateral prefrontal cortex	L	−38	50	6	3.4	254

*Cluster definition threshold: Z > 2.3; Cluster probability threshold: p < 0.05. Hem, hemisphere; L, left; R, right; B, bilateral; x, y, z, coordinates of local maxima in MNI space; Z, maximum Z statistic, k, cluster extent*.

### Total AQ score is associated with increased activation to biological>scrambled motion

**Figure 2:**
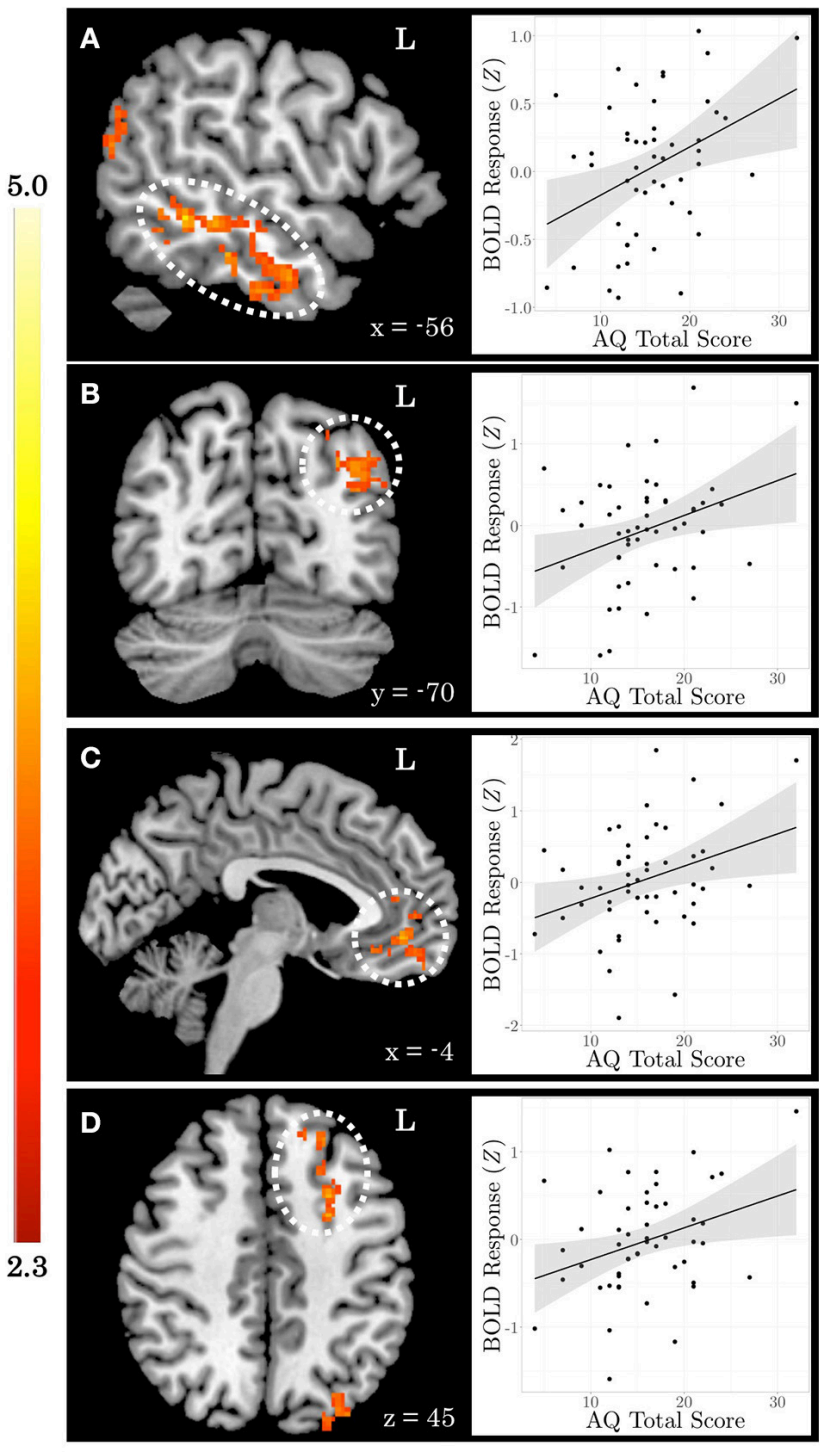
Autistic-like traits in healthy adults are associated with increased neural response during biological motion perception. On the left, *Z* statistic maps showing significant clusters of activation are depicted in MNI space. On the right, mean *Z* statistic values from each cluster are plotted against total Autism Quotient (AQ) score for each participant (*N* = 51). Gray shading indicates 95% confidence interval around best-fit line. **(A)** Temporal cortex. **(B)** Lateral occipital cortex. **(C)** Ventromedial prefrontal cortex. **(D)** Dorsolateral prefrontal cortex.

When testing for individual differences in the biological>scrambled motion contrast as a function of autistic-like traits, we find a positive association between total AQ score and neural activity in four clusters (Figure 2): (a) left temporal cortex extending from anterior inferior temporal gyrus to pSTS; (b) left lateral occipital cortex; (c) bilateral ventromedial PFC, extending from rostral PFC to anterior cingulate cortex (ACC); and (d) left dorsolateral PFC, spanning superior and middle frontal gyri. Table 3 contains local maxima statistics for this analysis. No clusters showed a significant negative relationship with total AQ score. Analyses testing for an interaction between total AQ score and sex yielded no significant clusters.

**Table 3:** Local maxima statistics for clusters showing a significant positive relationship with total AQ score.

**Anatomical region**	**Hem**	***x***	***y***	***z***	***Z***	***k***
Temporal cortex	L	−60	−32	−8	3.92	589
Lateral occipital cortex	L	−42	−74	30	3.68	431
Ventromedial prefrontal cortex	B	2	52	2	3.41	281
Dorsolateral prefrontal cortex	L	−26	12	42	3.31	225

*Cluster definition threshold: Z > 2.3; Cluster probability threshold: p < 0.05. Hem, hemisphere; L, left; x, y, z, coordinates of local maxima in MNI space; Z, maximum Z statistic, k, cluster extent*.

### Relationship between AQ subscores and activation to biological>scrambled motion

**Figure 3:**
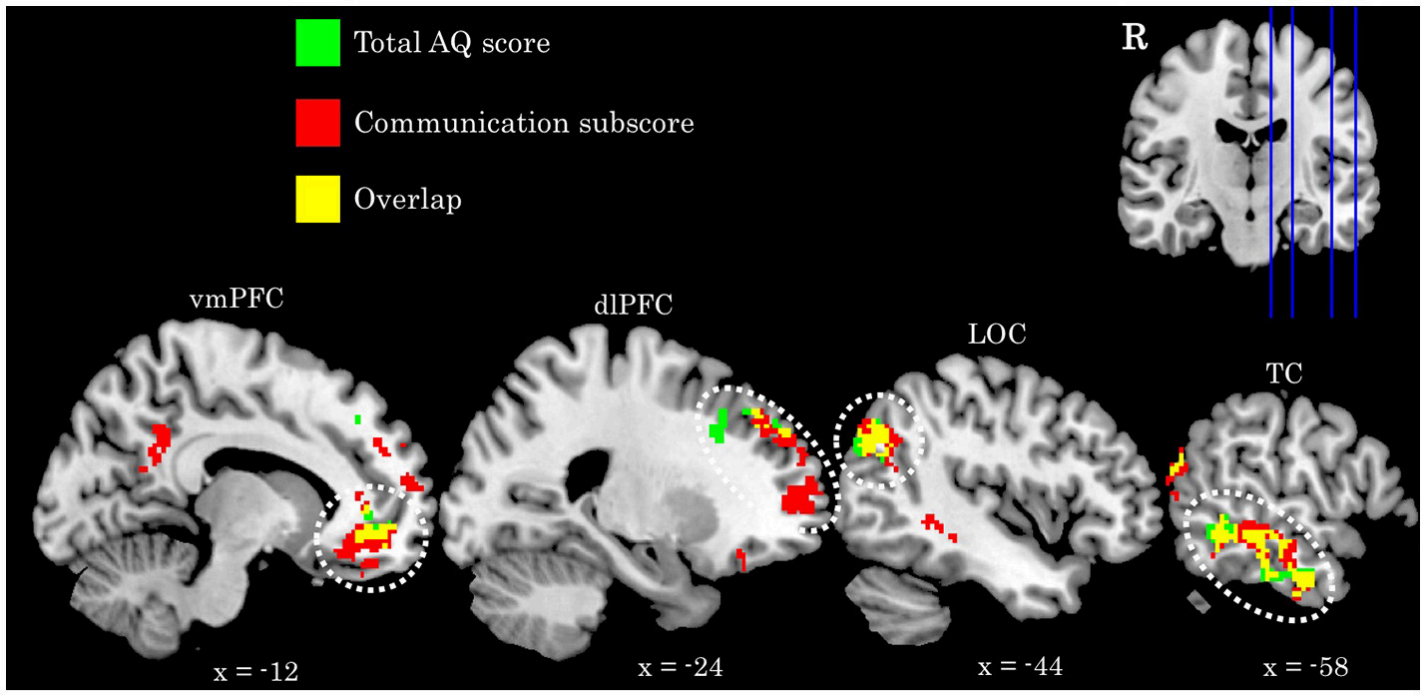
Comparison of activation identified in the total AQ score (green clusters) and Communication subscore (red clusters) analyses. Yellow voxels indicate those that overlap between these analyses. Image in upper left depicts location of sagittal slices. Coordinates are in MNI space. R, right; vmPFC, ventromedial prefrontal cortex; dlPFC, dorsolateral prefrontal cortex; LOC, lateral occipital cortex; TC, temporal cortex.

We find a positive association between AQ communication subscore and neural activity in five clusters: (a) left temporal cortex extending from anterior inferior temporal gyrus to pSTS; (b) bilateral ventromedial PFC extending into ACC; (c) rostral dorsolateral PFC; (d) left lateral occipital cortex; and (e) bilateral posterior cingulate cortex. Further examination of these clusters revealed a high degree of overlap between these regions and those identified in the total AQ score analysis. Figure 3 depicts regions of overlap between these analyses. Subscore analyses also revealed a positive relationship between social skill subscore and a cluster in right fusiform cortex extending into right cerebellum (crus I), and a negative relationship between imagination subscore and right supramarginal gyrus. These clusters did not overlap with those identified in the total AQ score analysis. No other subscores yielded significant results. Table 4 contains local maxima statistics for the subscore analyses.

**Table 4:** Local maxima statistics for clusters showing a significant association with AQ subscores.

**Subscore**	**Association**	**Anatomical region**	**Hem**	***x***	***y***	***z***	***Z***	***k***	**Overlap**
Communication	Pos	Temporal cortex	L	−60	−32	−8	4.35	785	71.31%
	Pos	Ventromedial PFC	B	−10	40	−8	4.34	1,228	86.83%
	Pos	Dorsolateral PFC	B	−22	56	8	3.75	557	22.67%
	Pos	Lateral occipital cortex	L	−50	−70	28	3.5	433	48.72%
	Pos	Posterior cingulate cortex	B	−14	−48	30	3.44	384	0.00%
Social skill	Pos	Fusiform cortex/Cerebellum (crus I)	R	28	−84	−26	3.36	239	0.00%
Imagination	Neg	Supramarginal gyrus	R	58	−42	48	3.6	266	0.00%

*Cluster definition threshold: Z > 2.3; Cluster probability threshold: p < 0.05. Hem, hemisphere; Pos, positive; Neg, negative; L, left; B, bilateral; x, y, z, coordinates of local maxima in MNI space; Z, maximum Z statistic, k, cluster extent; Overlap, percent of voxels within each cluster that overlaps with clusters identified in the total AQ analysis*.

## Discussion

This study shows that neural response during passive viewing of point-light displays of biological motion varies as a function of autistic-like traits assessed via the AQ. Individuals with more autistic-like traits show increased neural response to biological motion in prefrontal and left temporal cortices. By using a continuous measure of autistic-like traits coupled with an exploratory, whole-brain approach, we were able to assess the role of a network of brain regions not traditionally associated with biological motion perception in healthy adults. For example, right pSTS is often discussed as the area of functional specialization for biological motion stimuli (Allison et al., 2000), and is found in our examination of group activation as a whole. However, when including AQ score as a covariate, we find differential activation within pSTS localized to the left hemisphere.

As a whole, the clusters that emerge from the analysis of the main effect of viewing biological vs. scrambled motion are different from those that emerge when including AQ as a covariate in the analysis. Only 0.01 and 0.03% of voxels identified in the AQ covariate analysis overlap with the biological>scrambled and the biological<scrambled contrasts of the task main effect analysis, respectively. A targeted region of interest approach testing for relationships with AQ only within regions identified in the main effect analysis would have actually greatly reduced our ability to identify meaningful individual differences because this analysis specifically identifies those regions that show *similarities* across subjects. Rather, by including AQ as a covariate in the model of whole-brain activation, we are able to identify regions that show differential activation as a function of autistic-like traits. Furthermore, mean is included as an explanatory variable in the covariate model because we expect activation to vary as a function of task condition. Therefore, only regions in which the covariate accounts for significant additional variance in activation above and beyond that which is accounted for by the task contrast will emerge. While a covariate certainly can account for additional variance in regions identified in the main contrast, it is not surprising that regions not identified in the main contrast emerge in this analysis.

The regions that emerge in the AQ covariate analysis largely overlap with regions involved in understanding others' intentions and behaviors (Carrington and Bailey, 2009; Ames et al., 2011). For example, while right pSTS is robustly activated during biological motion perception (Allison et al., 2000), left pSTS is more sensitive to the *intent* of biological motion (Morris et al., 2008). Similarly, ventromedial PFC is implicated in understanding others' intentions and behaviors (Amodio and Frith, 2006; Gilbert et al., 2006; Frith, 2007), but is not typically discussed with biological motion perception in adults. The increased activation in these regions among individuals with more autistic-like traits might reflect greater inferential effort to understand the biological motion.

This interpretation is in line with a recent study that examined neural response to eye gaze as a function of autistic-like traits (Nummenmaa et al., 2012). Biological motion perception is not limited to the dynamics of whole body, but can include other forms of human motion that convey meaningful social information, such as eye movements (Allison et al., 2000). Neumann and colleagues presented participants with images of faces in which the eye gaze either remained constant or shifted from side, to direct, to side. They found that increased autistic-like traits were associated with increased neural response to shifted vs. constant eye gaze in regions that also emerge in the present study, including left temporal cortex and regions of prefrontal cortex. They interpret this increased activation within regions involved in inferring others' mental states to potentially reflect individual differences in the spontaneous tendency to draw inferences from eyes (Nummenmaa et al., 2012).

A similar hypothesis is further supported in the present study by the fact that the communication subscore of the AQ largely drives these effects, and also identifies an additional region associated with interpreting others' intention, the posterior cingulate cortex (Cavanna and Trimble, 2006; Carrington and Bailey, 2009). While defined as a communication domain, this subset of questions (e.g., “I find it easy to ‘read between the lines’ when someone is talking to me;” “I know how to tell if someone listening to me is getting bored”) largely touches upon one's ability to understand and appropriately interpret the mental states of their communication partner.

It has been hypothesized that the overarching function of the brain is to compute predictions based on prior experiences (Friston et al., 2009; Friston, 2010). Discounting information that is consistent with predictions reduces redundancy and results in lower activation (van Boxtel and Lu, 2013). Specifically, pSTS and regions of the action observation network, which includes temporal and prefrontal regions, are thought to play an anticipatory or predictive role in action perception (Rizzolatti and Sinigaglia, 2010; Vander Wyk et al., 2012). Consistent with this hypothesis, it has been shown that viewing highly unfamiliar actions (Cross et al., 2012) or motions that violate expectations (Brass et al., 2007; Jastorff et al., 2011) is associated with increased activity in prefrontal and temporal regions in adults.

Increased activity in similar regions in the present study might reflect a diminished ability to use prior experience with human action to accurately predict and interpret the current stimuli. Evidence from a recent biological motion adaptation study supports this hypothesis. Thurman et al. (2016) found increased autistic traits were associated with reduced perceptual aftereffects and diminished neural adaptation to biological motion in pSTS. A failure to adapt and desensitize to biological motion stimuli might reflect an inability to integrate prior perceptual experience with biological motion into the stored motion concept for use in subsequent predictions.

Sensitivity for biological motion increases throughout development—children reach adult levels of discrimination around middle adolescence (Hadad et al., 2011). At a neural level, young children automatically recruit PFC to a greater extent while simultaneously relying less on posterior regions during biological motion perception (Carter and Pelphrey, 2006) and understanding the intention of motion (Vander Wyk et al., 2012). With age, a shift in neural reorganization from frontal to posterior regions co-occurs with skill acquisition and is indicative of reduced effort and increased automatization (Johnson et al., 2009). The pattern of prefrontal neural activation associated with autistic-like traits in the present sample may therefore reflect a differential neurodevelopmental trajectory for individuals across the autistic-trait spectrum.

Anderson et al. (2013) specifically considered age-related differences in biological motion perception and its relation to non-clinical social deficits. They found that children and adolescents show a negative relationship between autistic traits and neural response in bilateral posterior and right frontal regions, and a positive relationship within left temporal regions. However, adults only showed a negative association between social deficits and neural response to biological motion perception. The present results in adults using a wider measure of autistic-like traits conflict with the adult data in this previous study. We see a positive relationship between autistic-like traits and neural activation in left temporal and prefrontal regions, which more closely aligns to their child population. One potential explanation for this discrepancy is that our sample is younger (18–25, *M* = 20.7 years) than the adults in this prior study (20–35, *M* = 24.7 years).

It is noteworthy that many studies that failed to find differences in biological motion perception between autistics and controls used older populations. This suggests that older ASD individuals may have adopted alternative strategies to discriminate biological motion (van Boxtel and Lu, 2013) that would be reflected in the recruitment of different neural networks resulting from divergent neurodevelopmental trajectories across the autism continuum.

Such altered neurodevelopmental trajectories may be established by variation at the genetic level. Converging research suggests variable genetic load for ASD may differentially impact neural systems specialized for biological motion perception. For example, while diagnosed individuals show hypoactivation of pSTS and PFC, their siblings, who share similar genetics but do not display autistic traits themselves, show increased activation in these regions during biological motion perception relative to controls (Kaiser et al., 2010b). The authors interpret this distinct neural response to reflect a compensatory mechanism by which siblings over-recruit these regions to overcome their high genetic risk. Similar etiologic mechanisms are thought to contribute to ASD traits across the continuum (Robinson et al., 2011), and thus might account for the positive association between autistic traits and neural response in these regions in our non-clinical sample.

### Limitations and future directions

While we favor a neurodevelopmental account for the present results that is supported by prior findings of age-related changes in neural systems supporting biological motion perception, we are unable to directly test this hypothesis in the current adult sample. Future research should employ a longitudinal design across a wide age range to better understand how neurodevelopment of biological motion perception varies as a function of autistic traits across the lifespan.

We hypothesize that differences in brain response might be related to genetic factors such as those seen in unaffected siblings (Kaiser et al., 2010b). It is important to note, however, that a study using avatars depicting actions that were either congruent or incongruent with the actor's emotional cue failed to find compensatory activity in unaffected siblings (Ahmed and Vander Wyk, 2013). This discrepancy highlights the importance of considering task parameters, and suggests that identifying genes that account for variation in specific behaviors or endophenotypes as a strategy to better understand complex disorders, like autism. While we do not include genetic analyses in the current study, the overlapping regions identified by Kaiser and colleagues and the present study identifies neural response to point-light displays of biological motion as a target endophenotype for future genetic studies.

The present study only considered a self-report measure of autistic-like traits, but the social behavioral phenotype is complex and not limited to characteristics associated with autistic traits. Future work should consider additional components of the social behavioral phenotype, such as social anxiety and schizotypal personality traits and include both clinical and non-clinical populations to better assess relationships across the full trait continuum. To avoid potential limitations associated with self-report, future studies should also collect measures of overt behavior or informant report.

Finally, while we did not find an interaction between AQ score and sex differences in the present sample, we may have been underpowered to find such an association in a whole-brain analysis. Considering that autism and autistic traits occur at different rates across sexes (Baron-Cohen et al., 2001), and neural response to biological motion varies in a sex-dependent manner (Anderson et al., 2013), sex differences should specifically be targeted in future work.

## Conclusions

Our study reveals a relationship between autistic-like traits in healthy adults and individual differences in neural response while passively viewing biological motion. These results emphasize the need for continuous phenotypic measures and the identification of neural endophenotypes, and are directly in line with the research domain criteria (RDoC) identified as a priority by the National Institute of Mental Health (Insel et al., 2010; Cuthbert and Insel, 2013). Our quantitative pairing of brain and behavioral assessments in healthy adults has revealed a potential mechanistic target for future research into typical and atypical developmental processes. Future studies investigating both typical and disordered perceptual and cognitive abilities, particularly those investigating underlying biological mechanisms, should carefully consider individual variability across the full trait continuum.

## Author contributions

MP and JM designed the research. MP acquired and analyzed the data. MP wrote the paper with contributions and comments from JM.

### Conflict of interest statement

The authors declare that the research was conducted in the absence of any commercial or financial relationships that could be construed as a potential conflict of interest.
